# Sonographic machine-assisted recognition and tracking of B-lines in dogs: the SMARTDOG study

**DOI:** 10.3389/fvets.2025.1647547

**Published:** 2025-08-08

**Authors:** Aurélie Jourdan, Caroline Dania, Maxime Cambournac

**Affiliations:** ^1^Centre Hospitalier Vétérinaire Frégis, Paris, France; ^2^IVC Evidensia France, Courbevoie, France

**Keywords:** artificial intelligence, lung ultrasound, B-lines detection, canine pulmonary edema, point of care ultrasound (POCUS)

## Abstract

**Introduction:**

Cardiogenic pulmonary edema (CPE) is a serious complication of heart failure in dogs, commonly characterized by excess fluid within the lung interstitium and alveoli. Point-of-care ultrasound (POCUS) allows for the prompt identification of pulmonary alterations through the presence of B-lines. However, interpretation remains subjective and operator dependent. Artificial intelligence (AI) may offer standardized, real-time analysis, but its application in veterinary medicine is largely unexplored.

**Objective:**

To assess the performance of an AI-based ultrasound algorithm in detecting B-lines in dogs and to evaluate its agreement with manual quantification by experienced operators.

**Methods:**

In this prospective study conducted at a single center, 40 dogs were enrolled: 20 with suspected CPE and 20 healthy controls. CPE suspicion was based on respiratory distress, a left atrium-to-aorta ratio (La:Ao) ≥1.6, >3 B-lines per field at thoracic POCUS, and clinical improvement following furosemide administration. Lung ultrasound was performed according to the Vet BLUE protocol. Cine loops were analyzed using the Butterfly Auto B-line Counter and reviewed independently by two POCUS-trained clinicians, each blinded to the AI results and to the other's evaluation.

**Results:**

The AI algorithm failed to provide a B-line count in 14.2% of cineloops overall, with failures occurring in 11.8% of the suspected CPE group and 2.4% of the non-CPE group. Quantification failures were significantly more frequent in the suspected CPE group (OR 4.88; *p* < 0.0001). Intraclass correlation coefficients showed excellent agreement for B-line counts (ICC = 0.88) and strong concordance for pathological classification (>3 B-lines; ICC = 0.85) between operators and AI. AI accuracy compared to clinicians was 84 and 86%.

**Conclusion:**

The AI algorithm demonstrated excellent agreement with experienced operators both for precise B-line counting and for the classification of pathological lung patterns. These findings support the potential of AI as a valuable decision-support tool for detecting clinically relevant cardiogenic pulmonary edema in veterinary critical care.

## 1 Introduction

Cardiogenic pulmonary edema (CPE) is a life-threatening complication of left-sided heart failure in dogs. Early detection and rapid intervention are crucial to improving survival and clinical outcomes (1). Traditionally, thoracic radiographs have been considered as the gold standard for diagnosing CPE (1, 2). However, this method requires placing often severely dyspneic or suffocating patients in recumbency and temporarily interrupting oxygen support. These conditions can be highly stressful, increasing the risk of hypoxemia decompensation, worsening respiratory distress, and potentially leading to fatal outcomes (3). Additionally, repeated imaging increases the risk of radiation exposure for both animals and veterinary team. To overcome these limitations, lung ultrasonography has emerged as a promising alternative. It is a non-invasive, rapid, bedside diagnostic tool that eliminates radiation risks and minimizes patient stress (4, 5). Studies have shown that lung ultrasound has sensitivity and specificity comparable to those of thoracic radiography for diagnosing CPE in dogs (6, 7).

Resulting from left-sided heart failure, CPE leads to fluid accumulation in the lungs. Lung ultrasound can detect this fluid by identifying specific artifacts known as B-lines. B-lines are vertical, well-defined hyperechoic vertical lines emerging from the pleural surface that move in synchrony with respiration (8). The presence of more than three B-lines is considered abnormal and indicates alveolar-interstitial syndrome, with its severity correlating to the number of B-lines detected (9, 10).

However, detecting B-lines can be operator-dependent, with accuracy improving with experience (11, 12). This variability is particularly critical in emergency settings, where multiple clinicians with varying levels of expertise work in shifts. It underscores the need for enhanced diagnostic methods to reduce operator variability (13). A diagnostic tool that is reliable, accurate, and rapid is essential to ensure diagnostic consistency, support less experienced clinicians, and enable quicker, more confident decision-making.

In response to the diagnostic challenges posed by operator-dependent variability and the risk of interpretation errors in lung ultrasound, artificial intelligence (AI) has emerged in human medicine as a promising solution. AI systems might be capable of automatically detecting and quantifying B-lines in real time, including both isolated and confluent patterns, thereby reducing reliance on clinician experience (13–15). Several human studies have assessed the agreement between AI-generated and human obtained B-line counts by experienced operators, reporting variable results ranging from moderate to excellent correlation depending on the lung site evaluated and operator's expertise (15–20). These findings suggest a promising potential for AI-assisted B-line quantification, although further refinement is needed before widespread clinical implementation (15). Importantly, this level of concordance has been associated with improved diagnostic accuracy, reduced time to diagnosis, and earlier therapeutic intervention, factors that likely contribute to enhanced patient outcomes.

However, no veterinary studies to date have evaluated the real-time performance of AI systems for B-line detection or compared their output to that of experienced clinicians in canine patients. This highlights a critical gap in the literature and underscores the need to assess the feasibility and reliability of AI-assisted lung ultrasound in veterinary practice.

The primary objective of this study was to evaluate the real-time ability of a human medicine-derived AI ultrasound system to automatically detect and count B-lines in dogs. A secondary objective was to assess the agreement between AI-generated B-line counts and manual counts performed on cineloops by experienced veterinarians.

We hypothesized that the AI algorithm would accurately detect and quantify B-lines in dogs with suspected CPE, and that there would be strong concordance between automated and manual B-line counts in this population.

## 2 Materials and methods

### 2.1 Study population

This single-center, prospective, observational study was conducted at CHV Frégis, between November 16th, 2024, and March 30th, 2025. Canine patients presenting to the emergency department with suspected CPE were included only if they met all of the following criteria: (a) physical examination findings consistent with signs of L-CHF (respiratory rate > 40 breaths/min (excluding panting), increased respiratory effort and/or crackles on thoracic auscultation) (9); (b) more than 3 B- lines per field on point-of-care ultrasound (POCUS) at least at two sites (6); (c) enlarged La:Ao ratio short axis (≥1.6:1) (21, 22); (d) positive clinical response defined as a reduction in respiratory rate of at least 10 breaths per minute within 6 h following furosemide administration; this 6-h timeframe was arbitrarily chosen to allow for early assessment of treatment efficacy. During this period, concurrent treatments including oxygen therapy and sedation were administered at the discretion of the emergency clinician.

Patients were excluded if a complete POCUS could not be performed due to hemodynamic instability, lack of cooperation with the probe. An additional group of healthy canine patients without respiratory signs was enrolled as a control group. They were deemed healthy based on their comprehensive medical history (no history of cardiac or respiratory disease), normal clinical examination, normal ultrasound findings including a La:Ao ratio < 1.6:1, and < 3 B-lines on point-of-care ultrasound.

### 2.2 Study design overview

#### 2.2.1 Sample size calculation

Sample size and power analyses were conducted to ensure adequate statistical power for assessing the agreement between three raters (Operator 1, Operator 2, and AI) using the Intraclass Correlation Coefficient (ICC). The analysis aimed to achieve 80% power at a significance level (α) of 0.05. We assumed an expected ICC of 0.85 and a null hypothesis ICC of 0.7, based on values reported in previous human studies (16, 17, 19, 20). Based on these parameters, and with 3 raters and 8 repetitions per dog already planned, the required sample size to achieve 80% power was determined to be 40 dogs.

#### 2.2.2 Cineloops acquisition

Point-of-care lung ultrasound was performed on each dog by a single POCUS-trained emergency and critical care resident who was not blinded due to his involvement in the clinical management of the animal. During the examination, a 6-s cine loop was captured at eight specific lung zones. At the time of acquisition, the AI algorithm automatically counted the number of B-lines, and this count was recorded. While the operator had access to the AI counting, patient management was guided exclusively by clinical expertise and bedside POCUS interpretation, independent of automated analysis.

#### 2.2.3 Cineloops evaluation and review

All cine loops were anonymously stored on a dedicated cloud platform created for this study.

The blinded review and manual B-line counting were performed simultaneously by both the POCUS-trained ECC resident and an experienced ECVECC specialist during a dedicated, non-clinical session conducted after image acquisition. To minimize potential bias, cine loops were randomized prior to review by renaming each file using a computer-generated random number system. Quality control was performed subjectively by the reviewers at the time of assessment. Loops where the pleural line was not clearly visible or those significantly affected by motion artifacts were considered non-interpretable and excluded from the analysis. This exclusion was done independently and in a blinded manner, meaning one reviewer could exclude a cine loop while the other accepted it, and vice versa. These excluded loops were not included in the B-line analysis, and the AI counts for these loops were disregarded. Both reviewers were blinded to the AI results and to each other's evaluations; manual counts were recorded and later compared to the automated AI-based counts.

### 2.3 POCUS protocol

**Figure 1:**
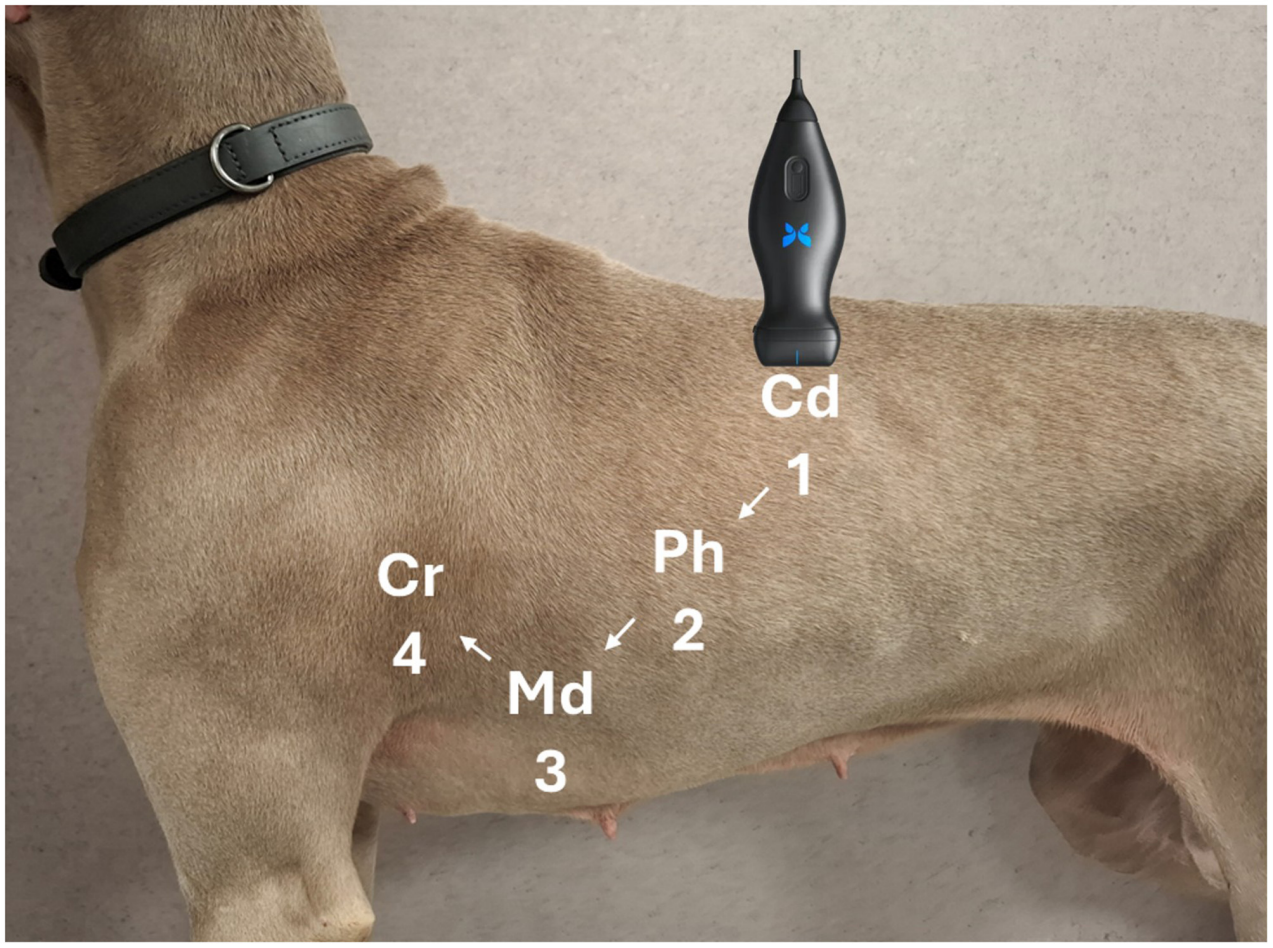
Representation of lung ultrasound probe positioning during the POCUS examination.

Eight acoustic windows were evaluated based on the Vet BLUE protocol, following a validated and consistent methodology (9, 23). Scanning was initiated at the caudodorsal lung region (Cd), corresponding to the TFAST chest tube site (CTS) in the 8th−9th intercostal space in the upper third of the thorax. The probe was then moved cranially through the perihilar (Ph) region (6th−7th intercostal space, middle third), the middle lung (Md) region (4th−5th intercostal space, lower third), and finally to the cranial lung (Cr) region (2nd−3rd intercostal space, lower third) (Figure 1). This sequence was repeated for the opposite hemithorax.

Before scanning, 70% isopropyl alcohol was applied to the fur (without clipping), and the dog was positioned in whichever stance it tolerated best, either standing (sternal) or in lateral recumbency.

All examinations employed a single handheld ultrasound-on-chip probe (Butterfly iQ; Butterfly Network Inc., Guilford, CT, USA). This device integrates ~9,000 capacitive micromachined ultrasonic transducer (CMUT) elements and beamforming electronics on a silicon substrate, providing a continuous bandwidth of 1–12 MHz. A veterinary-specific software interface allows the operator to select imaging presets on-screen, avoiding the need to swap physical transducers (24). For pulmonary imaging, the “Lung” preset was chosen. Upon activation, the CMUT aperture configures itself electronically as a convex array (3–5 MHz) to penetrate depths up to roughly 15 cm, enabling detection of artifacts such as B-lines (25). During each window, the operator adjusted gain and depth to center the pleural line on the screen, facilitating B-line detection and clear visualization of the lung field. The AI-driven system enforced a minimum depth of 8 cm. The probe was held perpendicular to the long axis of the ribs within the selected intercostal space, with the pleural line centered, to identify the pulmonary–pleural interface. This approach aligns with the standard POCUS protocol used routinely in clinical practice.

### 2.4 B-lines quantification

An artificial intelligence-human-based algorithm (the Auto B-line Counter, Butterfly Network) was used for automated B-line counting.

**Figure 2:**
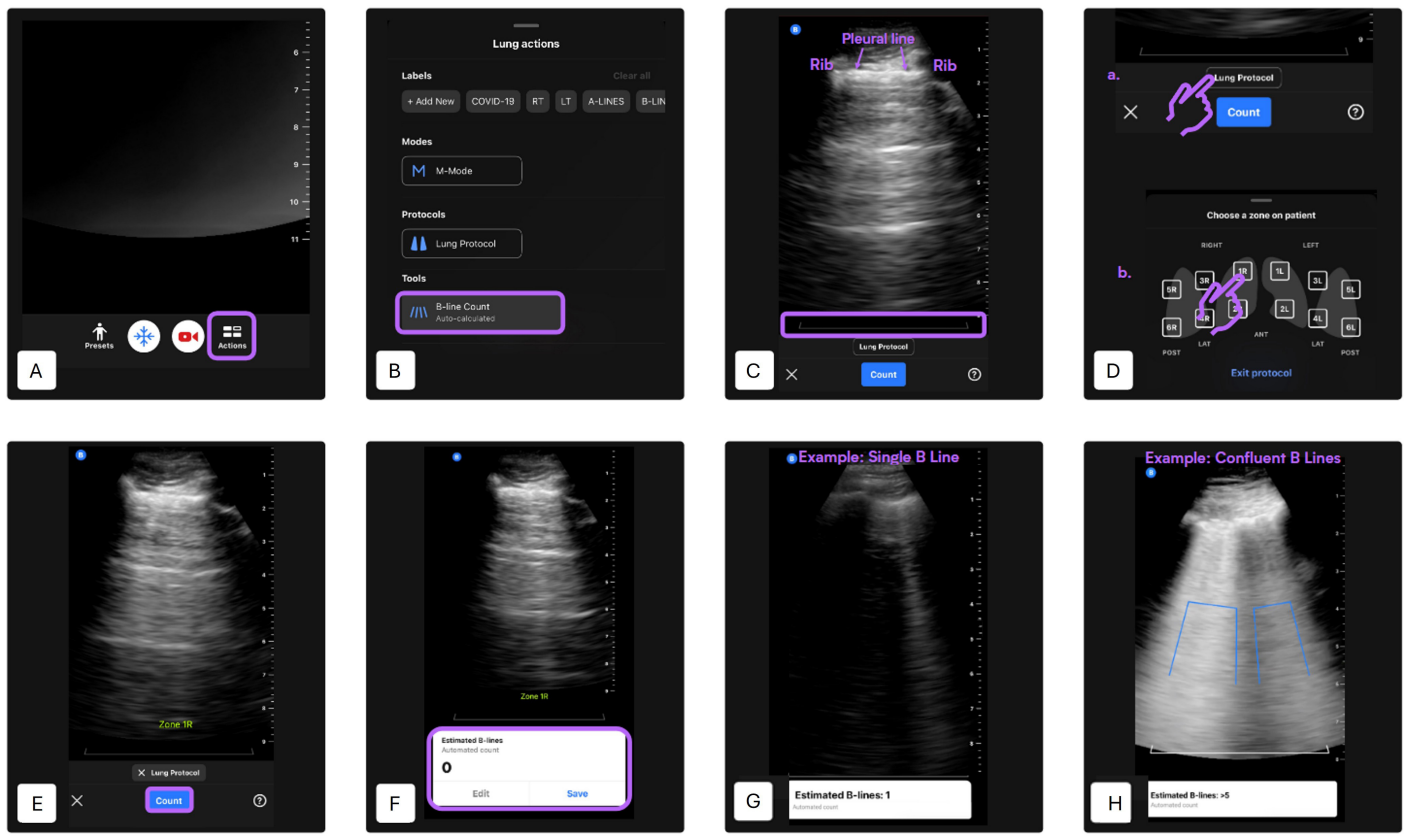
Automated B-line counting procedure using the Lung preset [modified from (27)]. **(A)** Lung preset selection—Begin by selecting the “Lung” preset from the main ultrasound interface. Tap the “Actions” button located at the bottom of the screen. **(B)** Tool selection—Under the “Tools” menu, select “B-Line Count” to activate the AI-based quantification tool. **(C)** Probe positioning—Place the probe in the desired intercostal space. Ensure that two ribs and the pleural line between them are clearly visible. Adjust gain and depth settings as necessary. A gray reference line will appear at the bottom of the image to indicate the level at which B-lines are assessed. The minimum scan depth is set at 8 cm. **(D)** Lung zone selection—Activate the lung protocol and select the lung zone being scanned. The selected zone label will appear on the screen. In this study, four lung quadrants were predefined per hemithorax: quadrant 1 is caudodorsal, quadrant 2 is perihilar, quadrant 3 is middle lung, quadrant 4 is cranial lung. **(E)** Initiate B-line detection—Press “Count” to begin automated B-line detection. A 6-s cine loop will be recorded automatically. Hold the probe as still as possible during acquisition. **(F)** Result display and clip management—If the AI successfully analyzes the clip, the estimated B-line count (ranging from 0 to >5) will be shown at the bottom of the screen. You can choose to “Save” the clip or “Edit” it. To scan additional zones, repeat steps **A, B, C, D** and **E** accordingly. **(G)** Example: single B-line detection—This panel shows a typical result of the AI identifying a single B-line. The B-line is highlighted as a vertical blue line extending from the pleural line. **(H)** Example: confluent B-lines—The AI detects multiple confluent B-lines. The vertical blue area outlines the coalescence of lines, representing an increased B-line score in this region.

B-lines were defined as laser-like vertical hyperechoic reverberation artifacts which arise from the pleural line, extend to the bottom of the screen without fading and move synchronously with lung sliding (10). This algorithm automatically provided the maximum number of B-lines visible within a single frame of a cine loop. The device indicated whether the automated B-line count was successful, and the number of attempts required to obtain a result was recorded. Counts ranged from 0, 1, 2, 3, 4, to >5. B-lines were highlighted on the captured cine loop using blue lines: a single line represented a discrete B-line, while a bracket indicated confluent B-lines (Butterfly Auto B-line Counter). The full procedure is illustrated below (Figure 2).

### 2.5 Statistical methods

Descriptive statistics were used to summarize the demographic characteristics of the study population. Continuous variables (age, body weight) are presented as median values, along with their minimum and maximum values and interquartile range (IQR: 25th−75th percentiles).

Comparative analyses between the healthy control and CPE groups were performed using non-parametric methods. Specifically, the Mann–Whitney U test was employed to assess differences in age and body weight. A *p*-value < 0.05 was considered statistically significant.

For performance assessment of the AI algorithm in B-line quantification, repetition rates and the proportion of algorithm failures were calculated. A mixed-effects logistic regression model was used to compute the odds ratio (OR) and evaluate the likelihood of failure in the suspected CPE group compared to controls. Age and body weight were initially included in the multivariable regression model to control for potential confounding. However, neither variable was significantly associated with the outcome, and both were excluded from the final model through stepwise backward elimination.

For the detection of pathological status based on B-line count (defined as >3 B-lines), a semi-quantitative analysis was performed. Agreement between raters was assessed using correlation coefficients, along with calculation of positive predictive value (PPV), negative predictive value (NPV), and overall agreement. The misclassification rate between operators and the AI was also determined. In addition, the accuracy of the AI in identifying pathological cases was calculated relative to each human operator.

To evaluate the consistency between raters, intraclass correlation coefficients (ICC) of type (1, 2) were calculated using a two-way random-effects model assessing absolute agreement. This analysis was conducted on both the raw B-line counts and the derived pathological classifications.

Finally, concordance analyses were conducted to explore cases of agreement or disagreement among the three evaluators (Operator 1, Operator 2, and AI).

All statistical analyses were performed using GraphPad Prism version 10.0.0 for Windows (GraphPad Software, Boston, MA, USA) and IBM SPSS Statistics version 29.0 (IBM Corp., Armonk, NY, USA), depending on the specific test employed.

## 3 Results

### 3.1 Demographic description

In the healthy group, the median age was 8.0 years (min 1.2, max 16.2; interquartile range [IQR]: 4.7–10.4 years). The median body weight was 20.4 kg (min 2.8, max 72; IQR: 8.5–33.0 kg). Breeds included: 3 Cavalier King Charles Spaniels, 2 mixed-breed dogs, 2 Labrador Retrievers, and one each of Golden Retriever, Cocker Spaniel, Griffon, Great Dane, American Staffordshire Terrier, Boxer, Pekingese, Dachshund, French Bulldog, Miniature Pinscher, Shiba Inu, German Shepherd, and Chihuahua.

In the suspected CPE group, the median age was 11.5 years (min 7.3, max 14.8; IQR: 10.2–12.8 years), and the median body weight was 6.7 kg (min 1.9, max 10.9; IQR: 5.0–8.6 kg). Breeds included: 7 Cavalier King Charles Spaniels, 5 Chihuahuas, 2 mixed-breed dogs, 2 Bichon Maltais, and one each of Pekingese, Toy Poodle, Shih Tzu, and French Bulldog.

Dogs in the suspected CPE group were significantly older (*p* < 0.001) and significantly lighter (*p* < 0.002) than dogs in the healthy control group. These variables were tested in the regression models as potential confounders but were not significantly associated with the outcome and were excluded from the final model.

### 3.2 Repetition rate and artificial intelligence performance

The AI ultimately succeeded in providing a B-line count in 100% of the dogs included in the study.

In the healthy group, 6 dogs required repeated attempts for successful B-line quantification by the AI algorithm. A total of 9 additional attempts were performed: 3 in a German Shepherd, 2 in a Golden Retriever, and 1 each in a Cocker Spaniel, Great Dane, Pekingese, and a mixed-breed dog. In 70% of healthy dogs, the AI provided a B-line count on the first attempt, 20% on the second attempt, and 10% after three or more attempts.

In the suspected CPE group, 9 dogs required repeated scans, with a total of 44 repetitions. These included one Cavalier King Charles Spaniel who required 3 attempts, and multiple Chihuahuas with up to 13 repetitions in one individual. A Shih Tzu required 7 attempts, and a Bichon Maltais required one additional attempt. In dogs with suspected cardiogenic pulmonary edema, the AI delivered a result on the first attempt in 55% of cases, after two attempts in 10%, and required three or more than three attempts in 35% of cases.

The AI algorithm failed to provide a B-line count at the first attempt in 14.2% of cineloops overall, with failures occurring in 11.8% of the suspected CPE group and 2.4% of the non-CPE group. Dogs with suspected CPE were significantly more likely to experience algorithm failures compared to healthy controls (odds ratio [OR]: 4.88; *p* < 0.0001).

### 3.3 B-lines count correlation and agreement

Of the 320 cine loops reviewed, 4 (1.2%) were considered uninterpretable by Operator 1 and 6 (1.9%) by Operator 2 due to poor video quality. These clips were excluded from the analysis.

When assessing diagnostic reliability, the ICC for absolute B-line count was 0.88 among Operator 1, Operator 2, and the AI algorithm. For the binary pathological classification (more than three B-lines vs. three or fewer), the ICC was 0.85 among the same evaluators.

Using the human operator's assessment as the reference standard, the AI algorithm demonstrated an accuracy of 84% compared to Operator 1 and 86% compared to Operator 2 in detecting pathological B-line counts.

For further comparison, Operator 1—the most experienced clinician—was designated as the gold standard. Compared to this reference, Operator 2 had a positive predictive value of 78.8%, a negative predictive value of 92.2%, and a misclassification rate of 11.4%. The AI algorithm, in comparison with Operator 1, showed a positive predictive value of 75.0%, a negative predictive value of 86.3%, and a misclassification rate of 16.1%.

Regarding the concordance of results:

- There were 0 instances where both operators agreed, but the AI differed.
- There were 0 instances where Operator 2 and the AI agreed, but Operator 1 differed.
- There were 3 instances where Operator 1 and the AI agreed, but Operator 2 differed.

## 4 Discussion

This study evaluates the real-time performance of a human-trained AI algorithm for B-line quantification in dogs with suspected CPE, directly comparing its accuracy with that of experienced veterinary clinicians. While absolute B-line quantification remains technically demanding and subject to interoperator variability, our findings highlight the potential clinical value of AI assistance, particularly when applying a clinically relevant threshold of >3 B-lines per field, a cutoff commonly used in both human and veterinary medicine to identify alveolar-interstitial syndromes such as CPE.

Raw B-line count absolute agreement between the AI algorithm and human operators were excellent (ICC = 0.88), consistent with and surprisingly surpassing, agreements reported in human-focused studies (15–17, 20). When a binary classification threshold was applied (>3 B-lines per field as pathological; ≤ 3 as physiological), the agreement between AI and human assessments remained excellent (ICC = 0.85), supporting the utility of this simplified approach for triage and decision-making. This aligns with recent human findings (18) and suggests that dichotomized interpretations may be equally robust and reliable as raw counts, particularly in emergency contexts where rapid, high-sensitivity screening is critical.

While these findings are promising, the slightly higher agreement observed in our veterinary data compared to human studies is consistent with our initial expectations. Several factors likely contributed to this outcome. First, the veterinary cohort was limited to a single pathological category (suspected cardiogenic pulmonary edema), while human studies (15–20) typically include a broader and more heterogeneous range of conditions such as cardiogenic pulmonary edema, acute respiratory distress syndrome (ARDS), COVID-associated pneumonia, or bronchopneumonia. This diagnostic homogeneity likely reduced interpretative variability and facilitated higher concordance. Furthermore, in our study, no alternative diagnoses (e.g., lung consolidations or shred signs suggestive of bronchopneumonia) were identified by either human operator, minimizing potential confusion between different vertical artifacts. While E-lines (from subcutaneous emphysema), I-lines (short, ill-defined vertical artifacts), and Z-lines (superficial, non-dynamic artifacts) can mimic true B-lines and may lead to AI misclassification (10), these were not predominant in our dataset. As such, artifact-related misinterpretation was unlikely to have significantly impacted AI-human agreement in the present analysis. However, this also highlights the need for future studies to include a wider spectrum of pulmonary diseases, in order to evaluate the AI's robustness in more diagnostically complex scenarios.

Second, although species-specific anatomical differences, such as thoracic conformation, lung volume, and the presence of fur, can complicate image acquisition, the relatively superficial location of the pleural line in dogs, along with the absence of obesity or thoracic deformities commonly encountered in human ICU patients, may have enhanced image clarity in many cases. Nonetheless, motion artifacts caused by spontaneous breathing in awake dogs may still have affected image quality. However, the use of experienced operators and standardized protocols likely mitigated these effects.

Third, although the sample size of our study was sufficient to reach 80% statistical power, it remained smaller than that of most human studies. This may have further limited variability and contributed to tighter agreement measures.

The AI algorithm demonstrated excellent diagnostic performance using human operators as the reference standard achieving an accuracy of 84–86%, with high negative predictive values (86.3–92.2%) moderate positive predictive values (75–78.8%) and a low overall misclassification rate (11.4–16.1%). This suggests that while the AI is effective at ruling out disease, it may overidentify pathology, resulting in false positives. Although conservative from a safety standpoint, this limited specificity raises concerns regarding its reliability in differentiating true pathological findings from benign artifacts. This highlights the critical need for further algorithmic refinement, particularly in adapting to the anatomical variability and unique disease presentations in veterinary patients, before the tool can be considered for routine clinical deployment. Without such optimization, the risk of overdiagnosis could undermine clinical confidence and lead to unnecessary interventions.

Notably, AI failures and the need for repeated cineloop acquisition were significantly more frequent in dogs with suspected CPE (OR = 4.88; *p* < 0.0001). This likely reflects the algorithm's difficulty in interpreting abnormal lung fields marked by pleural irregularities and confluent artifacts. These findings reinforce the importance of refining AI tools to distinguish clinically meaningful vertical artifacts from benign mimics. Additionally, dogs with suspected CPE were significantly smaller on average, which may have further complicated probe contact and acoustic window acquisition. These findings emphasize the importance of patient factors such as size, conformation, and respiratory effort, on both image acquisition and AI performance.

Technical variables also warrant attention, particularly probe positioning and orientation, which may directly influence AI performance. In small-breed dogs with narrow intercostal spaces, suboptimal probe contact or angulation may reduce image quality and alter the appearance of B-line artifacts, potentially affecting algorithmic detection and quantification. In this study, probe use was standardized according to the Vet BLUE protocol (23), involving longitudinal placement in the intercostal space with cranial marker orientation. However, alternative orientations such as transverse placement parallel to the ribs, may improve acoustic coupling and artifact visualization in small patients, possibly enhancing AI interpretation. While such strategies have been explored in human medicine (26), their impact on automated B-line detection in veterinary patients remains unknown and warrants further investigation.

Interobserver variability is a recognized challenge in lung ultrasound and may influence both manual and AI-assisted assessments. Although not directly evaluated in this study, previous research has demonstrated that agreement between clinicians can vary by thoracic region, with the highest reliability reported in anterior/superior zones (19). While results were not stratified by lung zone in the present analysis, such regional variability could contribute to discrepancies in B-line quantification and warrants consideration in future investigations.

This study has limitations. Echocardiographic confirmation of congestive heart failure (CHF) was not required for inclusion, which introduces a potential risk of misclassification. While many dogs likely had underlying cardiac disease, the possibility of concurrent or alternative respiratory conditions such as lower airway disease or pulmonary hypertension, both known to generate B-lines, cannot be excluded. That said, this potential misclassification may have limited impact on the core aim of the study. The objective was not to definitively diagnose cardiac heart failure but rather to identify dogs exhibiting B-lines, regardless of the underlying cause. In this context, even if some animals were incorrectly classified from a cardiogenic standpoint, they might still be appropriate for inclusion since they presented the pulmonary artifact (B-lines) under investigation. Importantly, no operator identified alternative artifacts or findings incompatible with B-lines, which reinforces the relevance of the collected data to the study's purpose. Additionally, the inclusion criteria based on compatible clinical signs, POCUS findings, and observed response to diuretic therapy, were designed to help mitigate this diagnostic uncertainty. Finally, the administration of concurrent treatments, such as oxygen therapy or sedation with butorphanol, may have influenced the observed clinical improvement. This could potentially confound interpretation of treatment response, although it does not detract from the core evaluation of AI performance in detecting B-lines. In addition, the AI system evaluated was developed and trained using human datasets, without prior adaptation to veterinary contexts. Its performance may therefore be affected by species-specific anatomical and physiological differences, including rib spacing, lung-to-chest wall ratios, and the presence of fur, which can interfere with probe contact and image acquisition. These factors likely contributed to discrepancies observed between algorithmic and manual assessments.

Future research should prioritize the development and validation of AI models trained on veterinary-specific datasets, encompassing a broad range of breeds, sizes, and clinical conditions. Larger, multicenter studies with varied operator experience and ultrasound platforms will also be essential to assess generalizability and integration into clinical workflows.

## 5 Conclusion

While B-line quantification can be technically demanding and subject to some inter-operator variation, our findings demonstrate excellent agreement between the AI algorithm and human evaluators—both in raw B-line counts and in binary classification (>3 B-lines per field). Using the most experienced clinician (Operator 1) as the reference, the AI algorithm achieved high negative predictive values (>85%) and moderately high positive predictive values (~75–80%), with misclassification rates ranging from around 15%. These results highlight the potential of AI-assisted protocols as effective triage or screening tools in emergency settings, where high sensitivity is critical. Although broader implementation is still constrained by species-specific anatomical differences and limited veterinary-specific training data, ongoing refinement and targeted validation could enable AI tools to enhance diagnostic accuracy, support clinical decisions, and expand access to point-of-care ultrasound in veterinary medicine.

## Data Availability

The original contributions presented in the study are included in the article/supplementary material, further inquiries can be directed to the corresponding author.
